# Three new physalins from *Physalis alkekengi* var. *franchetii*

**DOI:** 10.1007/s13659-013-0021-z

**Published:** 2013-05-31

**Authors:** Wan-Xuan Xu, Jian-Chao Chen, Jie-Qing Liu, Lin Zhou, Yi-Fen Wang, Ming-Hua Qiu

**Affiliations:** 1 State Key Laboratory of Phytochemistry and Plant Resources in West China, Kunming Institute of Botany, Chinese Academy of Sciences, Kunming, 650201 China; 2 University of Chinese Academy of Sciences, Beijing, 100049 China

**Keywords:** *Physalis alkekengi* var. *franchetii*, steroid, physalin, neophysalin

## Abstract

Three new physalin steroids, physalin III (**1**), physalin IV (**2**), 3-*O*-methylphysalin X (**3**), together with five known physalins (**4–8**) were isolated from the 80% EtOH extract of calyces of *Physalis alkekengi* var. *franchetii*. The structures of the new compounds were revealed through 1D and 2D NMR and mass spectroscopic studies.

**Electronic Supplementary Material:**

Supplementary material is available for this article at 10.1007/s13659-013-0021-z and is accessible for authorized users.

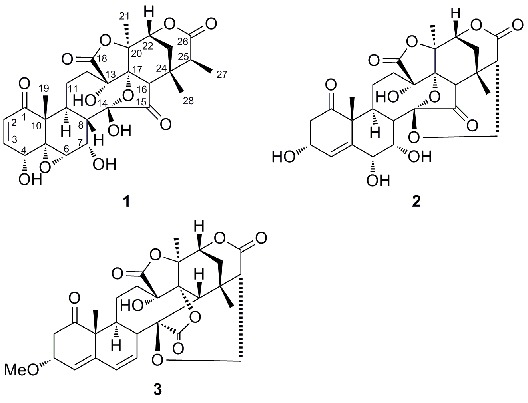

## Introduction

The genus of *Physalis* (Solanaceae), including about 120 species, is widely distributed throughout South and North America, which also includes five species, and two variations which also can be found in China[1]. The plant *Physalis alkekengi* var. *franchetii*, is used in traditional Chinese medicine[2], and is chiefly used for the treatment and prevention of tumors, leishmaniasis, sore throat, cough, eczema, hepatitis and urinary problems[3]. The chemical constituents of *P. alkekengi* var. *franchetii* are mainly composed of alkaloids, flavonoids, sterols, fatty acids, amino acids, and steroids[4].

Physalins, as one of the characteristic constituents from *P. alkekengi* var. *franchetii*, is a type of steroids with 16,24-cyclo-13,14-*seco*-ergostane skeleton which has been established by X-ray crystallographic analysis[5–7]. Neophysalins, are another characteristic structure, and are transformed from physalins through an acid-induced benzilic acid-type rearrangement reaction[8]. Until recently, more than 50 physalins and neophysalins have been identified from *P. alkekegi var. franchetii*, *P. agulata*, *P. lancifolia* and *P. minima*. Physalins and neophysalins have tremendous biological activities, including anti-tumor[9], anti-microbial[10], anti-malarial[11], immunosuppressive[12], anti-inflammatory[13,14], immunomodulatory[15], cytotoxic[16], and trypanocidal[17,18] effects.

In this study, five physalins (**1**, **2**, **4–6**) and three neophysalins (**3**, **7**, **8**) were isolated from *P. alkekengi* var. *franchetii*, three of which (**1–3**) were new. Their chemical structures and their elucidation are described herein.

## Results and Discussion

Compound **1** was obtained as a white powder. The molecular formula of **1** was determined to be $C_{28}H_{32}O_{12}$ by HREIMS (*m/z* 560.1885, $[M]^+$; calcd 560.1894) and NMR data (Table 1). The $^1$H NMR spectrum of **1** (in pyridine-$d_5$) showed four methyl signals at $\delta_H$ 1.29, 1.47, 1.65, and 2.02, four oxygenated signals at $\delta_H$ 4.01 (H-4), 3.54 (H-6), 5.70 (H-7) and 4.73 (H-22), and two olefinic proton signals at $\delta_H$ 6.24 (H-2) and 7.19 (H-3). The $^{13}$C DEPT spectrum of **1** (in pyridine-$d_5$) displayed the presence of twenty-eight carbons, including four methyls, three methylenes, ten methines (two olefinic carbons and four oxymethanes), eleven quaternary carbons (two ketone groups, two lactone groups, four oxygenated carbons, and one ketal carbon). These signals indicated the structural similarity of **1** and physalin J[19]. The chemical shift of the ketal carbon at C-14 ($\delta_C$ 103.4) of **1** differently from that ($\delta_C$ 107.8) of physalin J, together with one more methyl signal and less an oxygenated $CH_2$ in **1** indicated that the C(27)-O-C(14) ether

*To whom correspondence should be addressed. E-mail: mhchiu@mail.kib.ac.cn

**Table 1. $^1$H NMR (600 MHz) and $^{13}$C DEPT (150 MHz) data of compounds 1–3**

| No. | physalin III (**1**)[a] | | physalin IV (**2**)[a] | | 3-*O*-methylphysalin X (**3**)[b] | |
|---|---|---|---|---|---|---|
| | $\delta_H$ (*J* in Hz) | $\delta_C$, type | $\delta_H$ (*J* in Hz) | $\delta_C$, type | $\delta_H$ (*J* in Hz) | $\delta_C$, type |
| 1 | | 202.9, C | | 212.3, C | | 213.8, C |
| 2 | 6.24, d (9.8) | 132.8, CH | 3.12, dd (11.7, 5.4); 2.85, dd (11.7, 5.7) | 47.1, $CH_2$ | 3.13, dd (13.4, 6.5); 2.62, m | 41.2, $CH_2$ |
| 3 | 7.19, dd (9.8, 6.5) | 145.6, CH | 4.67, dd (5.7, 5.4) | 67.7, CH | 4.42–4.38, m | 76.7, CH |
| 4 | 4.01, d (6.3) | 70.3, CH | 6.32, d (4.4) | 130.5, CH | 5.70, d (4.1) | 122.4, CH |
| 5 | | 66.2, C | | 145.8, C | | 142. 8, C |
| 6 | 3.54, br. s | 62.9, CH | 4.79, d (3.8) | 77.4, CH | 6.28, dd (10.4, 1.5) | 127.5, CH |
| 7 | 5.70, br. s | 65.4, CH | 5.23, dd (3.5, 1.6) | 70.6, CH | 6.17, dd (10.4, 3.4) | 127.9, CH |
| 8 | 2.93, m | 44.1, CH | 3.39, dd (11.7, 1.6) | 43.8, CH | 2.81, m | 47.7, CH |
| 9 | 3.45, m | 32.2, CH | 2.11, dd (11.7, 2.7) | 32.2, CH | 2.71–2.74, m | 32.8, CH |
| 10 | | 49.8, C | | 53.3, C | | 51.0, C |
| 11 | 2.89, m | 20.4, $CH_2$ | 1.63–1.54, m; 2.34, m | 23.9, $CH_2$ | 2.05, d (4.4); 1.71–1.59, m | 28.0, $CH_2$ |
| 12 | 2.47, m; 2.25–2.31, m | 32.1, $CH_2$ | 2.58, ddd (16.5, 13.0, 6.2); 1.95, dd (16.5, 9.4) | 26.8, $CH_2$ | 1.92, d (1.3); 2.04, m | 31.9, $CH_2$ |
| 13 | | 80.8, C | | 80.3, C | | 80.8, C |
| 14 | | 103.4, C | | 108.5, C | | 82.3, C |
| 15 | | 217.0, C | | 209.7, C | | 169.5, C |
| 16 | 3.23, s | 55.6, CH | 3.26, s | 55.3, CH | 2.60, s | 51.1, CH |
| 17 | | 83.6, C | | 81.9, C | | 83.9, C |
| 18 | | 173.5, C | | 173.7, C | | 173.3, C |
| 19 | 1.65, s | 15.3, $CH_3$ | 1.78, s | 19.8, $CH_3$ | 1.36, s | 21.2, $CH_3$ |
| 20 | | 83.9, C | | 82.6, C | | 82.5, C |
| 21 | 2.02, s | 22.3, $CH_3$ | 2.32, s | 22.9, $CH_3$ | 1.76, s | 22.3, $CH_3$ |
| 22 | 4.73, br. s | 77.6, CH | 4.75, m | 78.0, CH | 4.58, dd (4.4, 1.4) | 76.1, CH |
| 23 | 2.14–2.19, m; 1.92, d (14.9) | 27.5, $CH_2$ | 2.11, m | 33.1, $CH_2$ | 1.97, m | 33.0, $CH_2$ |
| 24 | | 36.0, C | | 32.0, C | | 29.7, C |
| 25 | 3.42, m | 42.4, CH | 3.05, d (3.8) | 51.2, CH | 2.57, dd (11.4, 2.3) | 41.7, CH |
| 26 | | 173.0, C | | 168.1, C | | 169.4, C |
| 27 | 1.29, d (7.5) | 17.5, $CH_3$ | 4.02, dd (13.4, 3.9); 4.83, dd (13.4, 4.5) | 62.4, $CH_2$ | 4.48, dd (12.7, 3.3); 4.32, m | 61.7 , $CH_2$ |
| 28 | 1.47, s | 26.4, $CH_3$ | 1.30, s | 26.1, $CH_3$ | 1.45, s | 31.7, $CH_3$ |
| OMe | | | | | 3.27, s | 55.7, $CH_3$ |

[a]in pyridine-$d_5$, [b]in $CDCl_3$.

bridge was ruptured. This was further determined by the HMBC correlations (Figure 1) from Me-27 ($\delta_H$ 1.29) to C-25 ($\delta_C$ 42.4) and C-26 ($\delta_C$ 173.0). Moreover, the HMBC correlations from $\delta_H$ 1.65 (H-19), 6.24 (H-2), and 7.19 (H-3) to $\delta_C$ 202.9 (C-1) demonstrated the presence of an conjugated 2-en-1-one group, and the $^1$H-$^1$H COSY correlations of $\delta_H$ 7.19 (H-3)/$\delta_H$ 4.01 (H-4) revealed that there existed an hydroxyl group at C-4. An oxymethine in **1** instead of a methene in physalin J suggests that one hydroxyl group could be located at C-7, which was confirmed by the $^1$H-$^1$H COSY correlations of $\delta_H$ 3.54 (H-6)/$\delta_H$ 5.70 (H-7) and $\delta_H$ 5.70 (H-7)/$\delta_H$ 2.93 (H-8). The relative configurations of H-8 and Me-28 in the physalin skeleton were established to be $\beta$-oriented by X-ray.[20,21] Thus, the NOE correlations (Figure 1) of H-4/H-6, H-6/H-7, H-7/H-8$\beta$, and H-27/Me-28 established the relative configuration of OH-4 and OH-7 to be $\alpha$-oriented and Me-27 to be $\beta$-oriented. Hence, the structure of compound **1** was identified to be physalin III (**1**).

**Figure 1.** Key HMBC (H→C) and ROESY(H ↔ H) correlations of compound **1**

Compound **2** was obtained as a colorless needle crystal. The molecular formula of **2** was determined to be $C_{28}H_{32}O_{12}$ on the basis of HREIMS (*m/z* 560.1854, $[M]^+$; calcd 560.1894) and NMR data (Table 1). The NMR data of **2** were similar to those of physalin Z[22], and the differences between them were two of the double-bond carbon signals in physalin Z were replaced by two oxygenated methine signals in **2** located at C-6 and C-7, which were deduced by $^1$H-$^1$H COSY correlations of H-8 ($\delta_H$ 3.39)/H-7 ($\delta_H$ 5.23) and H-7 ($\delta_H$ 5.23)/H-6 ($\delta_H$ 4.79) and by HMBC correlations from H-6 to C-10, C-4, C-8, C-7, and C-5, from H-7 to C-6, C-5, C-8, and C-14. The ROESY correlations (Figure 2) of H-6/H-7 and H-7/H-8$\beta$ revealed that OH-C(6) and OH-C(7) were $\alpha$-oriented. So the structure of compound **2** was deduced and named as physalin IV (**2**).

**Figure 2.** Key HMBC (H→C) and ROESY(H ↔ H) correlations of compound **2**

Compound **3** was obtained as an amorphous yellow powder. The molecular formula of **3** was determined as $C_{29}H_{32}O_{10}$ using HREIMS and NMR data (Table 1). These NMR data of **3** were similar to those of physalin X[23], except the presence of an additional methoxyl group. Comparing with physalin X, the chemical shift of C-3 ($\delta_C$ 76.7) at down field and the HMBC correlation (Figure 3) of OMe ($\delta_H$ 3.27) with C-3 in **3** indicated a methoxyl group at C-3. The relative configuration of Me-19 and H-16 in physalin skeleton were established to be $\beta$-oriented by X-ray.[20,21] So the relative configuration of 3-OMe could be determined as $\alpha$-oriented by the NOE correlations of Me-19/H-3 and H-16/H-3. Hence, the structure of compound **3** was identified to be 3-*O*-methylphysalin X (**3**).

The structures of five known steroidal compounds, physalin Z (**4**)[22], physalin C (**5**)[24], physalin I (**6**)[25], physalin P (**7**)[26], 25,27-dihydro-4,7-dedehydro-7-deoxyneophysalin A (**8**)[27], were elucidated in comparison to their spectroscopic data with those reported in the literature.

**Figure 3.** Key HMBC (H→C) and ROESY(H ←→ H) correlations of compound **3**

## Experimental Section

**General Experimental Procedures.** Column chromatography (CC): silica gel (100–200 mesh, 200–300 mesh; Qingdao Marine Chemical Co., Ltd.), Sephadex LH-20 (Pharmacia). Thin-layer chromatography (TLC): silica gel $60F_{254}$ (Qingdao Marine Chemical Co., Ltd.). Optical rotations: Jasco P-1020 polarimeter. UV Spectra: Shimadzu UV-2401 PC spectraphotometer. IR Spectra: Bruker Tensor-27 infrared spectrophotometer, with KBr pellets in $cm^{-1}$. NMR Spectra: Bruker Bruker Avance III-600MHz spectrometer; chemical shifts $\delta$ were recorded as *ppm* relative to TMS, coupling constants $J$ in Hz. ESIMS: API QSTAR Pulsar spectrometer; HREIMS: Waters Autospec Premier P776.

**Plant Material.** The calyces of *Phsalis alkekengi* var. *franchetii* were purchased in Kunming CTM market, Kunming city, Yunnan province at December 2011 and were identified by Prof. Xi-Wen Li who was botanic taxonomist in Kunming Institute of Botany.

**Extraction and Isolation.** Dried calyces (15 kg) of *P. alkekengi* var. *franchetii* were extracted with 80% EtOH under reflux. The extract was suspended in water (5 L) and then partitioned successively with PE (petroleum ether) (8 L × 4) and EtOAc (8 L × 5). The EtOAc-soluble layer (evaporated under vacuum to get 250 g) eluted successively with $CHCl_3$, $CHCl_3$-MeOH (100:1), $CHCl_3$-MeOH (20:1), and $CHCl_3$-MeOH (5:1). Then, the $CHCl_3$-MeOH (100:1) portion (80 g) was chromatographed on a silica gel column using a gradient of $CHCl_3$-$CH_3OH$ (100:0 to 0:100), which yielded five fractions (1–5). Fraction 2 was successively subjected to RP-18, Sephadex LH-20 and silica gel to derive subfractions I–IV. Subfraction II was further separated over silica gel with $CHCl_3$-$CH_3OH$ (120:1) to yield compound **3** (5 mg), **6** (4 mg), **7** (10 mg) and **8** (3 mg). Subfraction III was subjected to semi-preparative HPLC ($CH_3CN$-$H_2O$, 50:50) to produce **4** (1 mg) and **5** (1 mg). Fraction 3 was successively subjected to RP-18, Sephadex LH-20 and separated over silica gel with $CHCl_3$-$CH_3OH$ (80:1) to yield **1** (2 mg) and **2** (6 mg).

**Physalin III (1):** white powder; $[\alpha]^{21}_{D}$ – 29.33 ($c$ 0.2, $CHCl_3$); UV ($CHCl_3$): $\lambda_{max}$ (log $\varepsilon$): 240 (3.36) nm; IR (KBr): $\nu_{max}$ 3430, 2924, 1787, 1758, 1728, 1462, 1376, 1218, 1073, 1041 $cm^{-1}$; $^1H$ and $^{13}C$ NMR: see Table 1. Negative ESIMS: 559 ([M – H]$^-$). HREIMS: 560.1885 [M]$^+$ (calcd. for $C_{28}H_{32}O_{12}$, 560.1894).

**Physalin IV (2):** colorless needle crystal ($CHCl_3$); $[\alpha]^{16}_{D}$ – 114.40 ($c$ 0.1, MeOH); UV (MeOH): $\lambda_{max}$ (log $\varepsilon$): 201 (4.23) nm; IR (KBr): $\nu_{max}$ 3431, 1781, 1729, 1632, 1383, 1374, 1167, 1134, 1065 $cm^{-1}$; $^1H$ and $^{13}C$ NMR: see Table 1. Positive ESIMS: 583 ([M + Na]$^+$). HREIMS: 560.1854 [M]$^+$ (calcd. for $C_{28}H_{32}O_{12}$, 560.1894).

**3-*O*-Methylphysalin X (3):** yellow powder; $[\alpha]^{18}_{D}$ – 16.00 ($c$ 0.1, pyridine); UV (MeOH): $\lambda_{max}$ (log $\varepsilon$): 225 (3.77), 201 (3.80) nm; IR (KBr): $\nu_{max}$ 3432, 2923, 1786, 1629 $cm^{-1}$; $^1H$ and $^{13}C$ NMR: see Table 1. Positive ESIMS: 563 ([M + Na]$^+$). HREIMS: 540.1933 [M]$^+$ (calcd. for $C_{29}H_{32}O_{10}$, 540.1995).

## Electronic Supplementary Material

Supplementary material is available in the online version of this article at http://dx.doi.org/10.1007/s13659-013-0021-z and is accessible for authorized users.

## Acknowledgments

This project was financially supported by the National Major Basic Research Program (No. SB2007FY400) of the National Ministry of Science and Technology of China, the National Knowledge Innovation Program of Chinese Academy of Sciences (No. KSCX2-YW-G-038), and the Foundation of State Key Laboratory of Phytochemistry and Plant Resources in West China (P2010-ZZ14).

**Open Access** This article is distributed under the terms of the Creative Commons Attribution License which permits any use, distribution, and reproduction in any medium, provided the original author(s) and source are credited.

## Electronic supplementary material

Supplementary material, approximately 1.54 MB.
