# Supplementary material for: Three new physalins from Physalis alkekengi var. franchetii
Source: Nat Prod Bioprospect. 2013 May 31;3(3):103–6. doi: 10.1007/s13659-013-0021-z (PMC4131671; doi:10.1007/s13659-013-0021-z)
Supplement: Supplementary file 1 — Supplementary material, approximately 1.54 MB. [file 13659_2013_21_MOESM1_ESM.pdf]

Electronic Supplementary Material

Three new physalins from *Physalis alkekengi* var. *franchetii*

Wan-Xuan XU,[a,b] Jian-Chao CHEN,[a] Jie-Qing LIU,[a] Lin ZHOU,[a] Yi-Fen WANG,[a] and Ming-Hua QIU[a,b,]*

[a]State Key Laboratory of Phytochemistry and Plant Resources in West China, Kunming Institute of Botany, Chinese Academy of Sciences, Kunming 650201, China

[b]University of Chinese Academy of Sciences, Beijing 100049, China

Received 10 March 2013; Accepted 3 May 2013

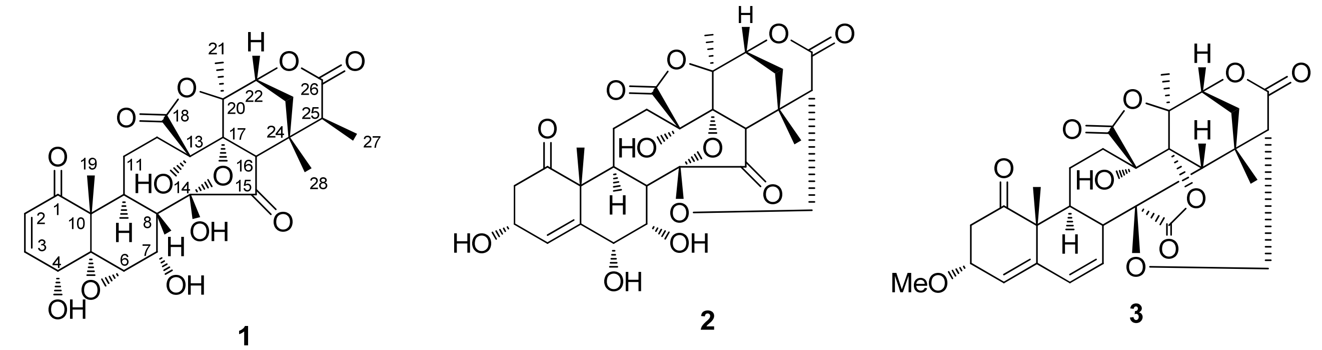

1 **2** **3**

Structures of compounds **1**–**3**

*To whom correspondence should be addressed. E-mail: mhchiu@mail.kib.ac.cn

Legends for Electronic Supplementary Material Figures

Fig. 1 ^{1}H NMR spectrum of compound **1** (pridine-d_5, 600 MHz).

Fig. 2 ^{13}C NMR spectrum of compound **1** (pridine-d_5, 150 MHz).

Fig. 3 HMBC spectrum of compound **1**.

Fig. 4 COSY spectrum of compound **1**.

Fig. 5 HSQC spectrum of compound **1**.

Fig. 6 ROESY spectrum of compound **1**.

Fig. 7 ^{1}H NMR spectrum of compound **2** (pridine-d_5, 600 MHz).

Fig. 8 ^{13}C NMR spectrum of compound **2** (pridine-d_5, 150 MHz).

Fig. 9 HMBC spectrum of compound **2**.

Fig. 10 COSY spectrum of compound **2**.

Fig. 11 HSQC spectrum of compound **2**.

Fig. 12 ROESY spectrum of compound **2**.

Fig. 13 ^{1}H NMR spectrum of compound **3** ($CDCl_3$, 600 MHz).

Fig. 14 ^{13}C NMR spectrum of compound **3** ($CDCl_3$, 150 MHz).

Fig. 15 HMBC spectrum of compound **3**.

Fig. 16 COSY spectrum of compound **3**.

Fig. 17 HSQC spectrum of compound **3**.

Fig. 18 ROESY spectrum of compound **3**.

Compound 1

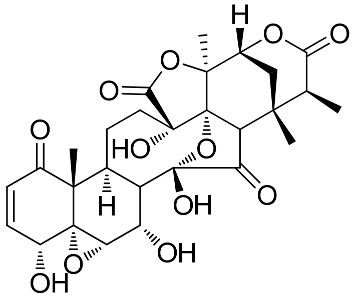

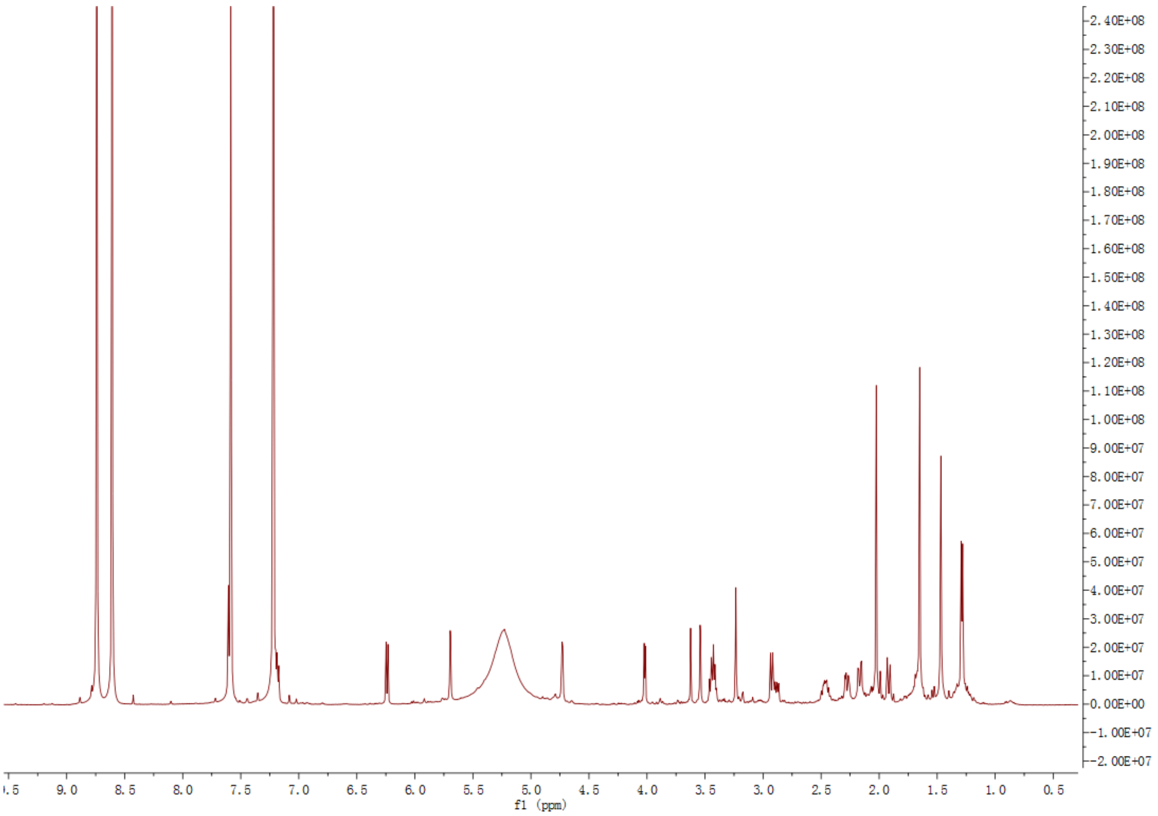

Fig. 1 ^{1}H NMR spectrum of compound **1** (pridine-d_5, 600 MHz).

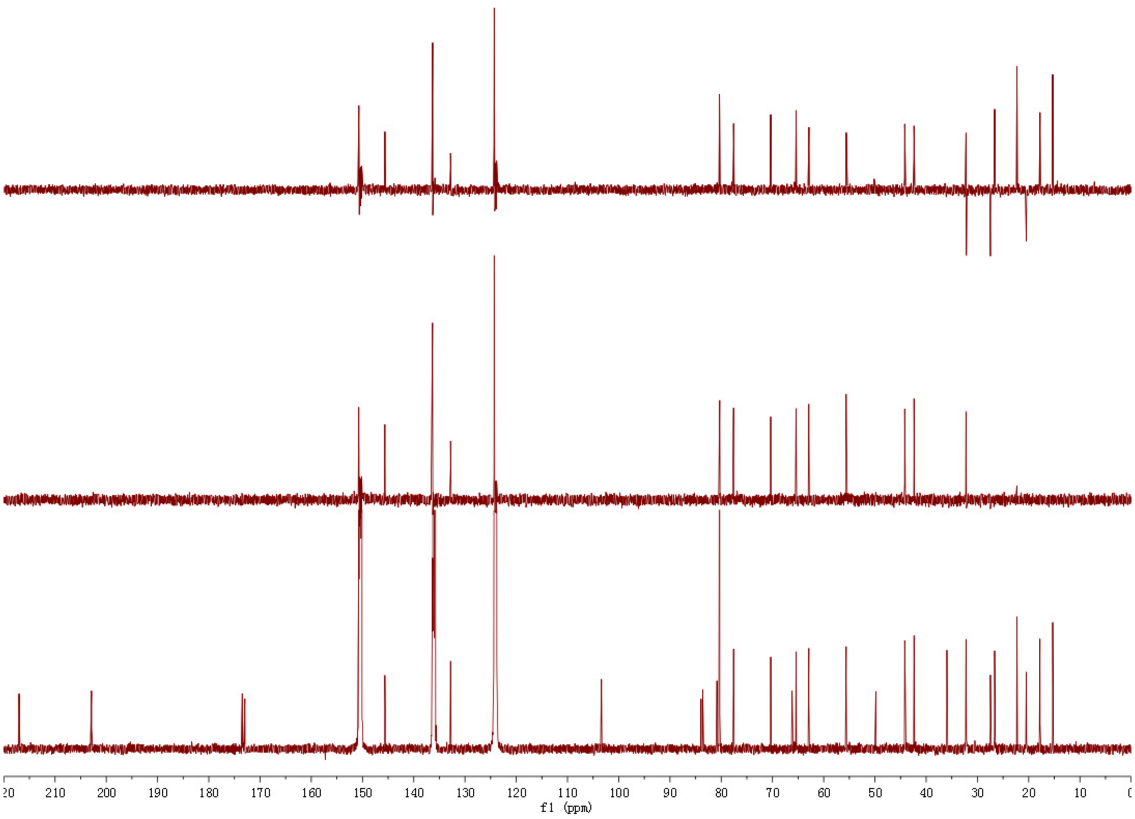

Fig. 2 ^{13}C NMR spectrum of compound **1** (pridine-d_5, 150 MHz).

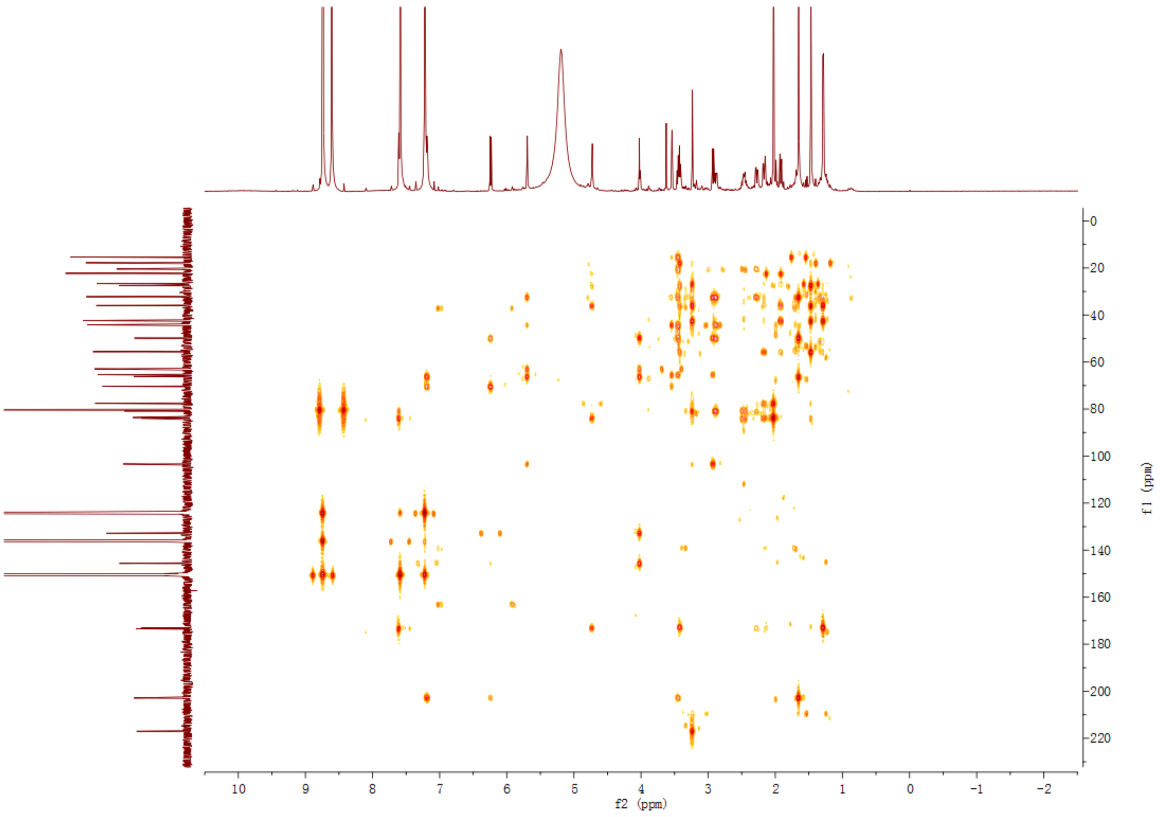

Fig. 3 HMBC spectrum of compound **1**.

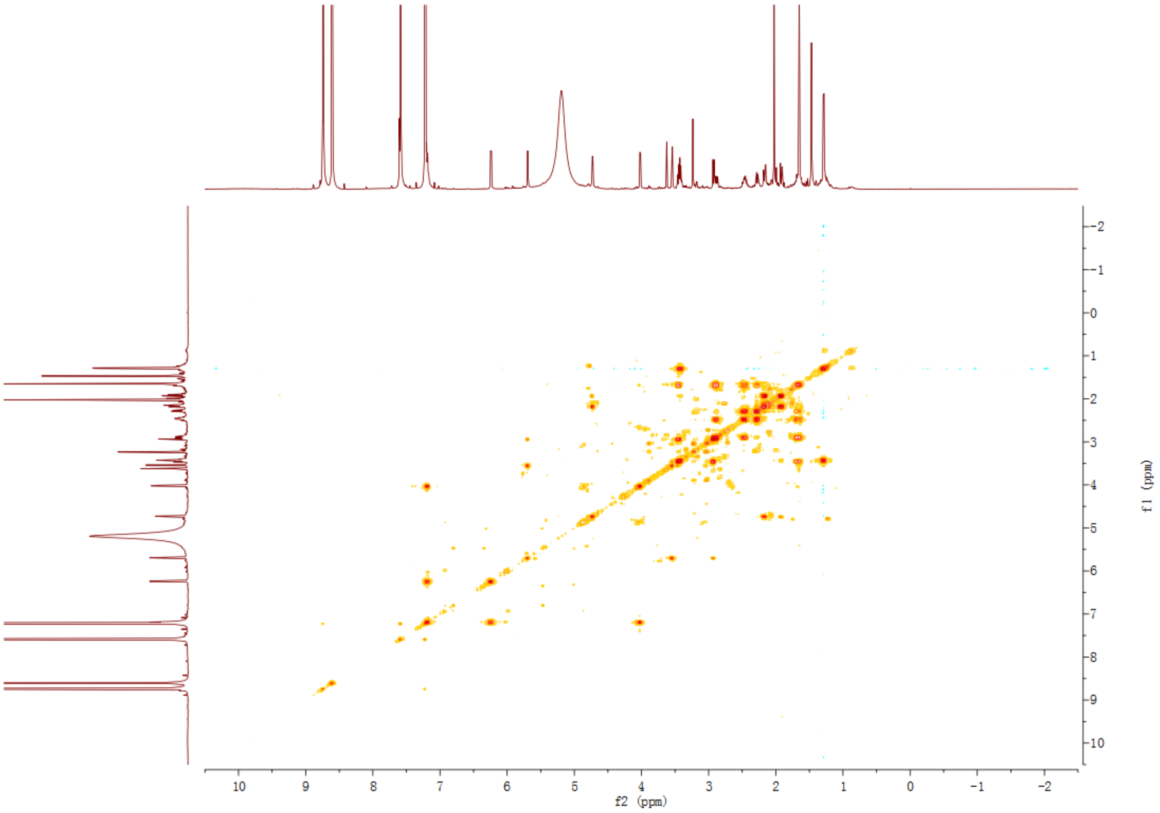

Fig. 4 COSY spectrum of compound **1**.

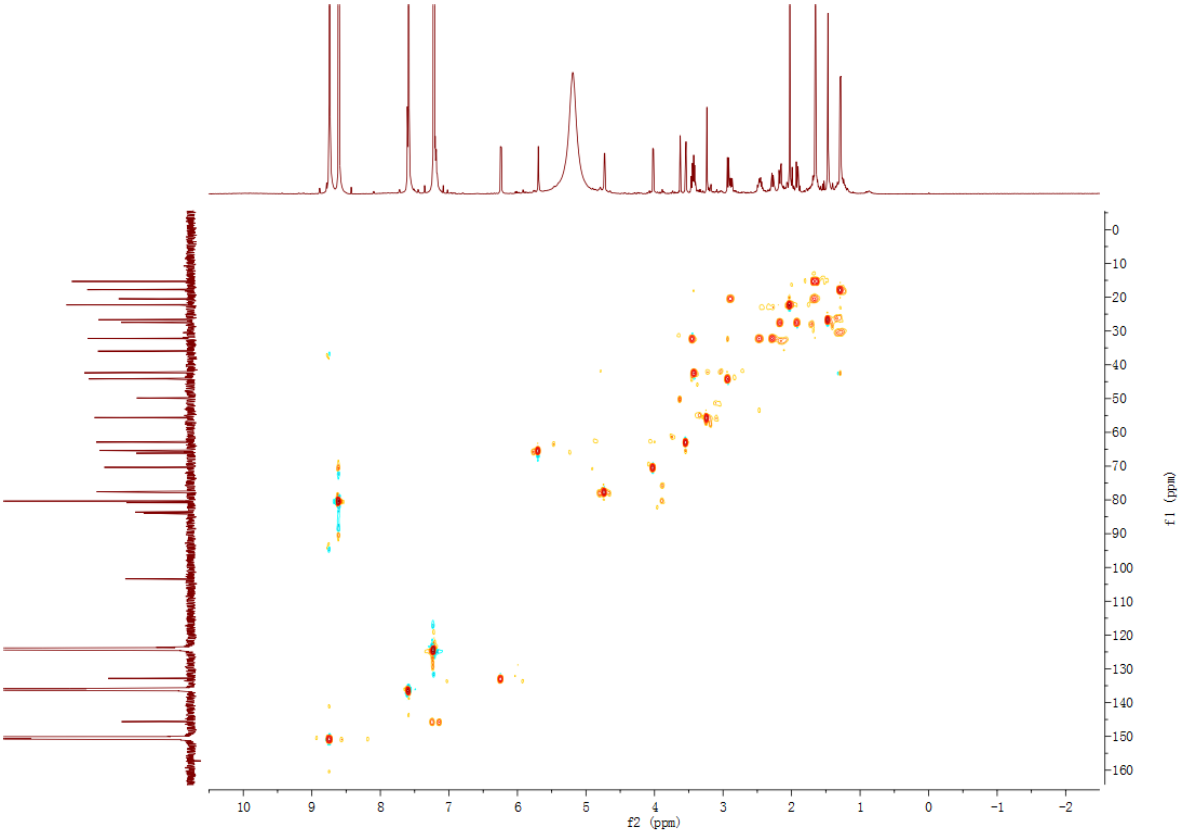

Fig. 5 HSQC spectrum of compound **1**.

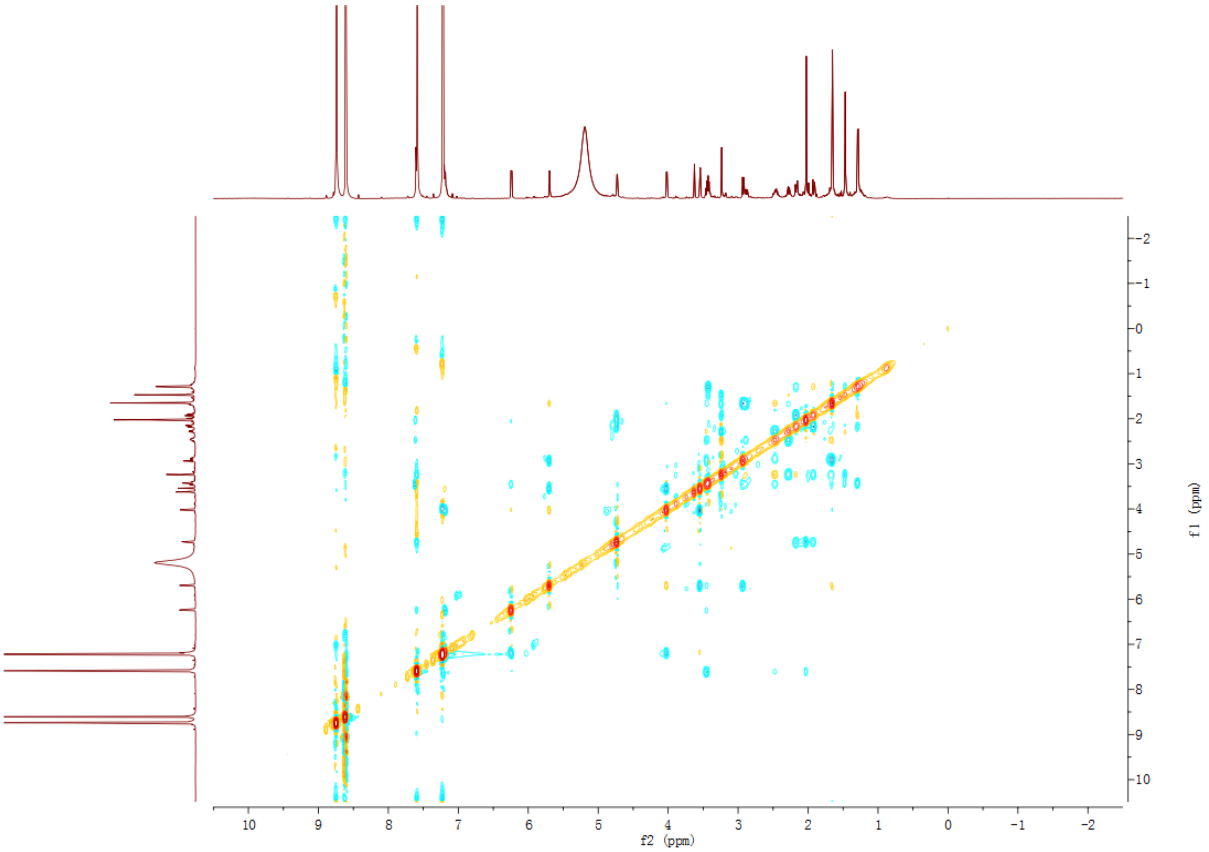

Fig. 6 ROESY spectrum of compound **1**.

Compound **2**

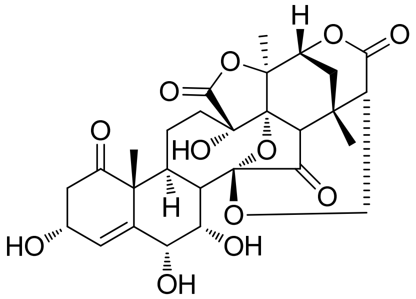

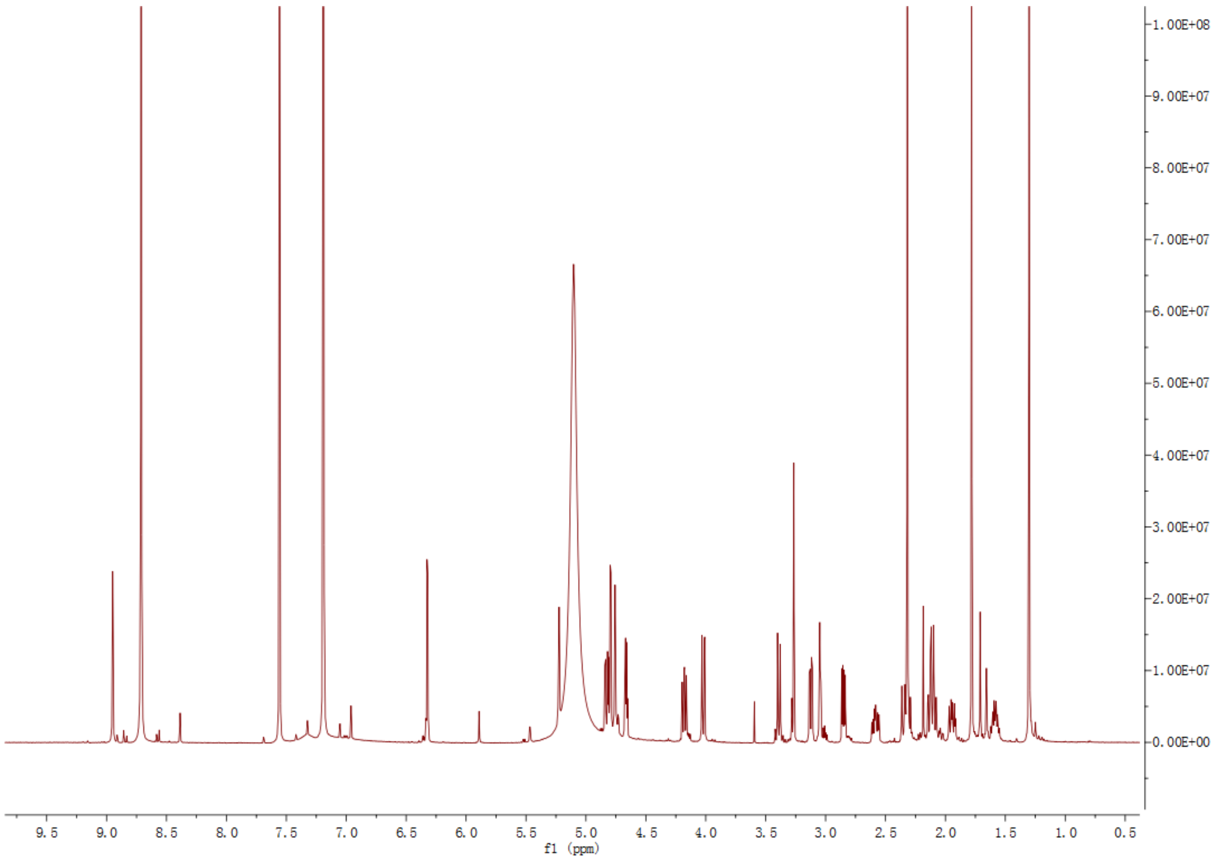

Fig. 7 ^{1}H NMR spectrum of compound **2** (pridine-d_5, 600 MHz).

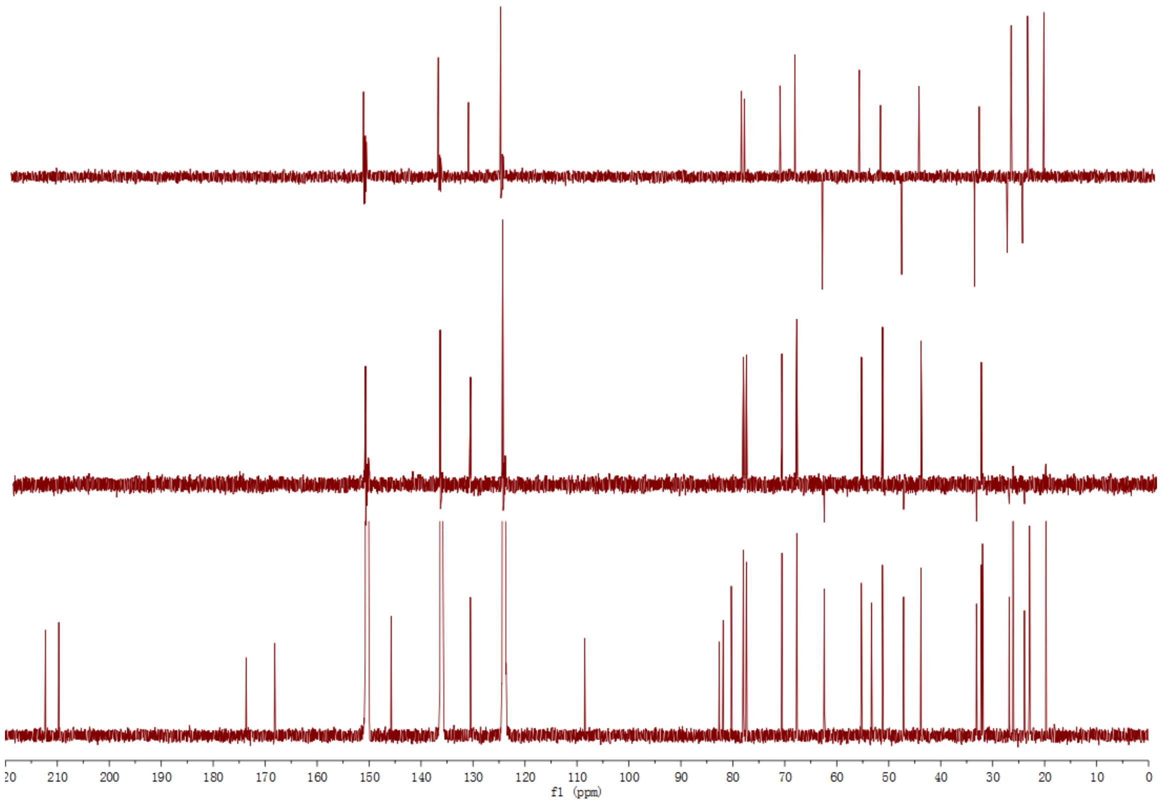

Fig. 8 ^{13}C NMR spectrum of compound **2** (pridine-d_5, 150 MHz).

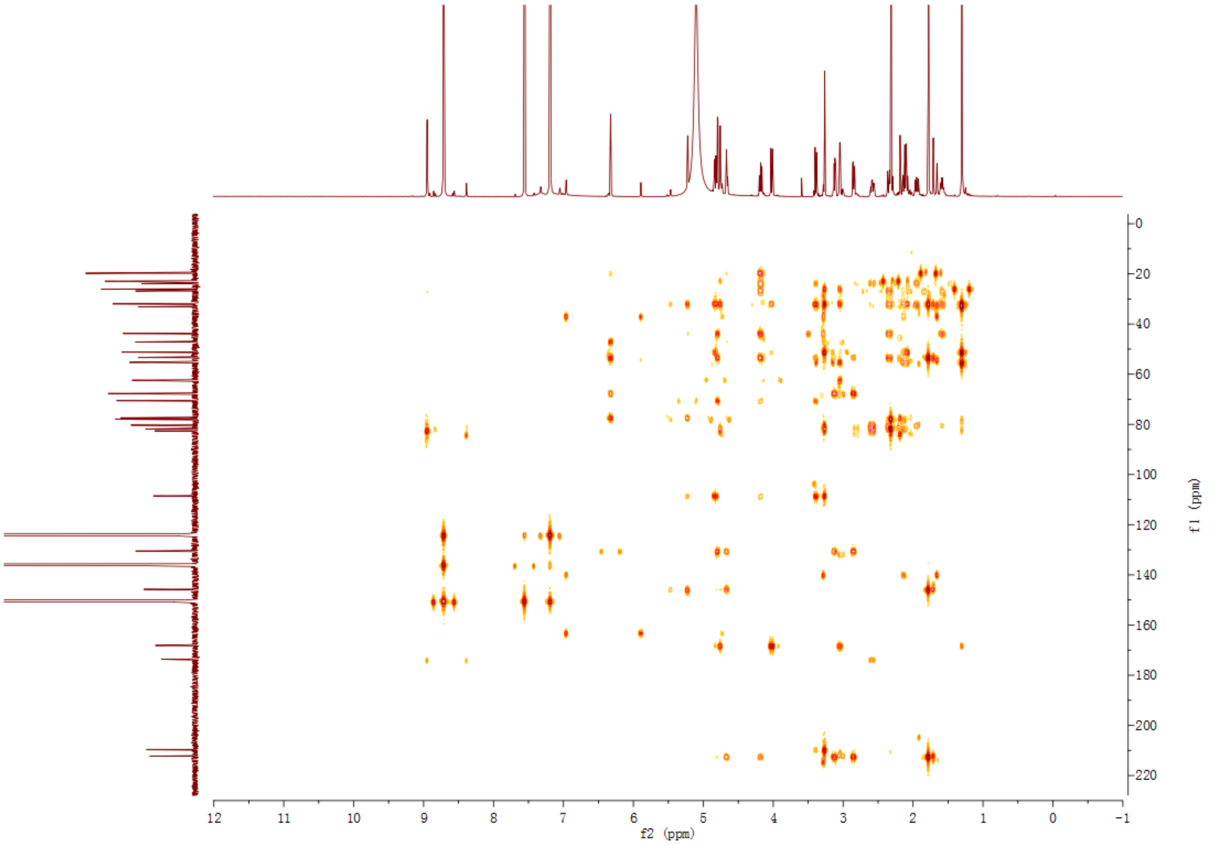

Fig. 9 HMBC spectrum of compound **2**.

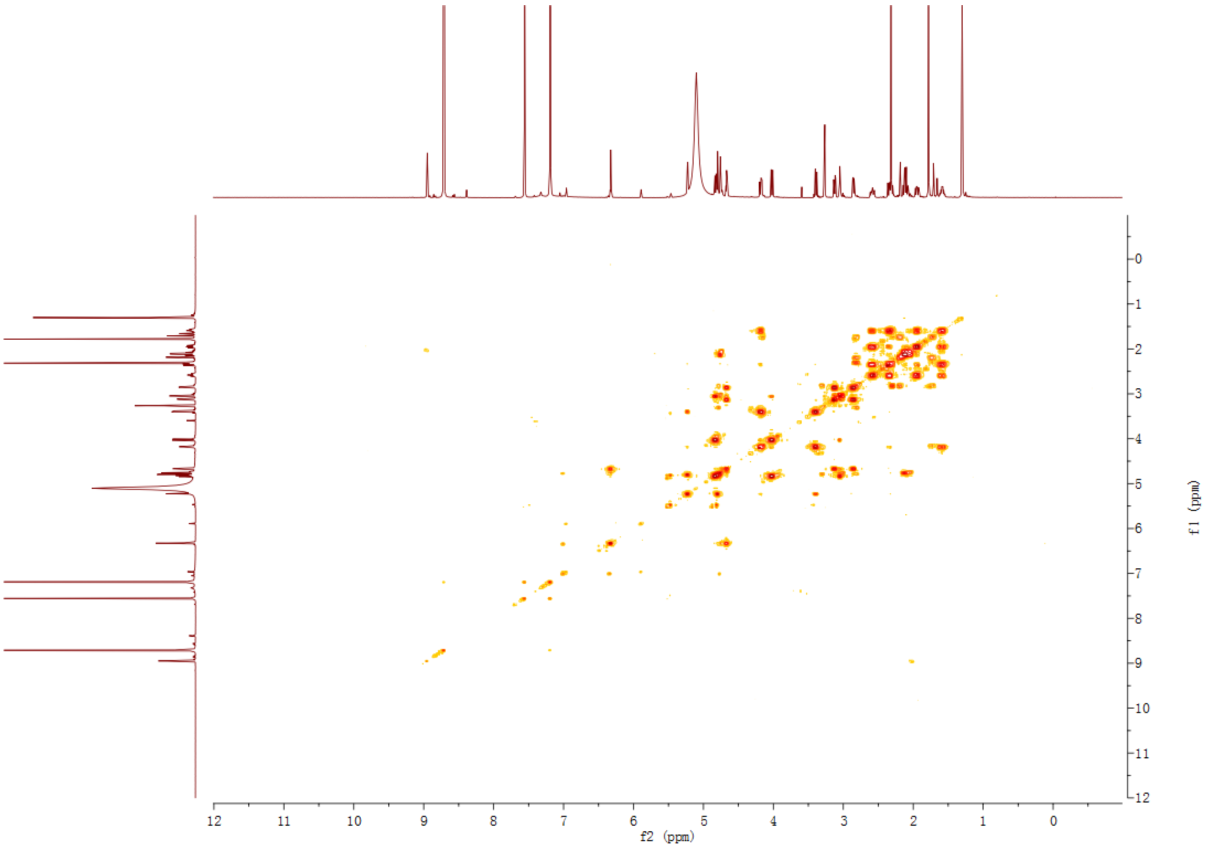

Fig. 10 COSY spectrum of compound **2**.

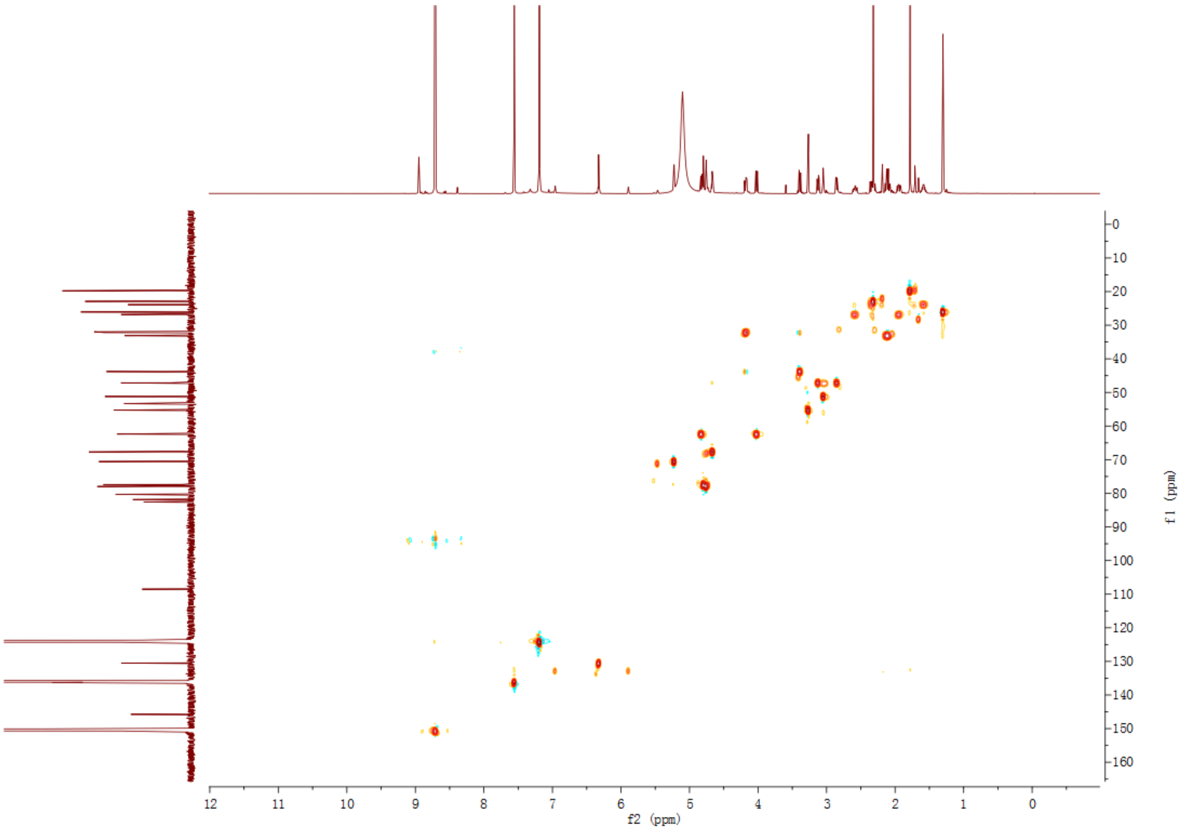

Fig. 11 HSQC spectrum of compound **2**.

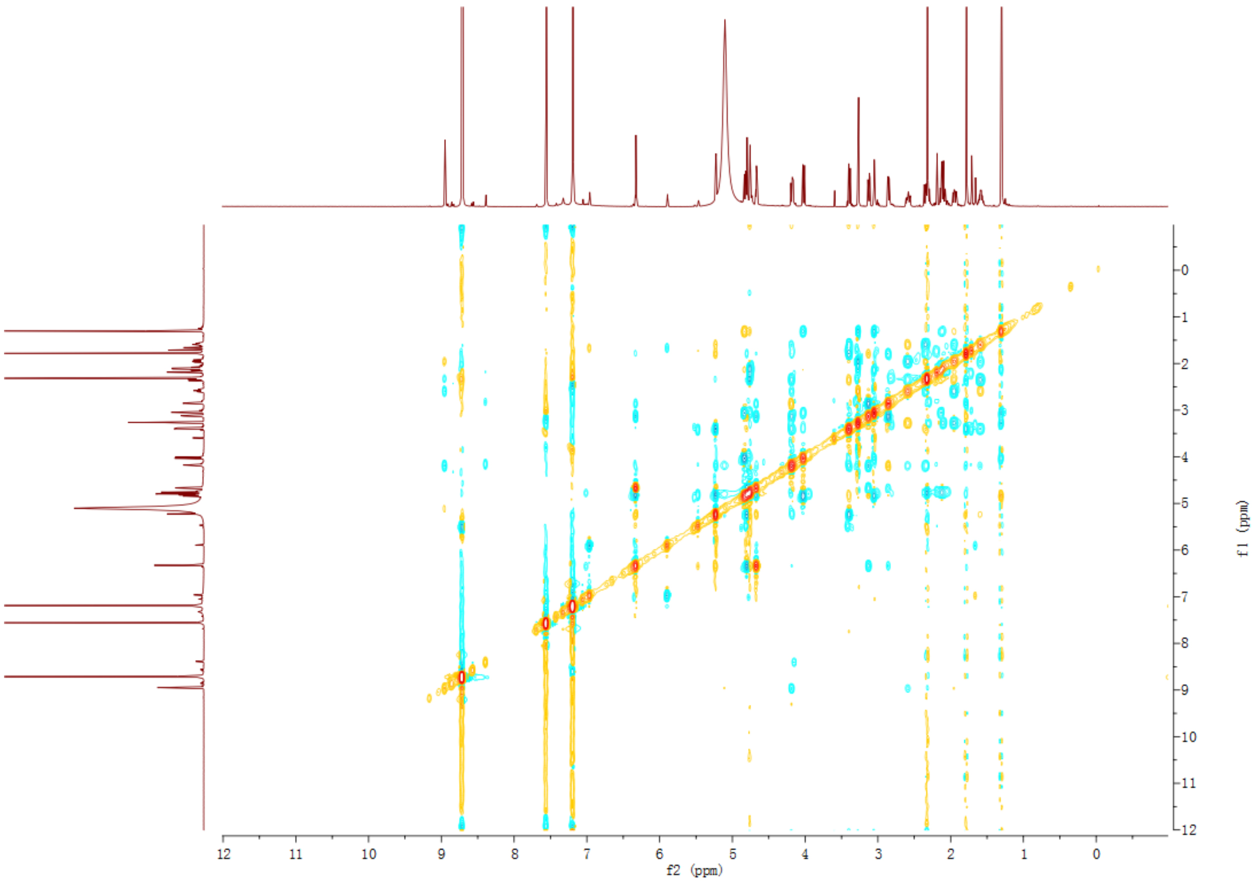

Fig. 12 ROESY spectrum of compound **2**.

Compound **3**

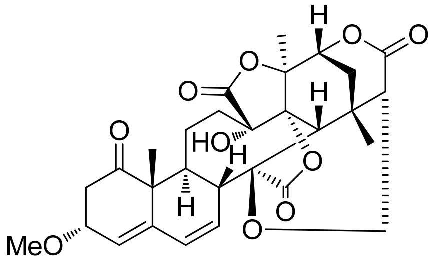

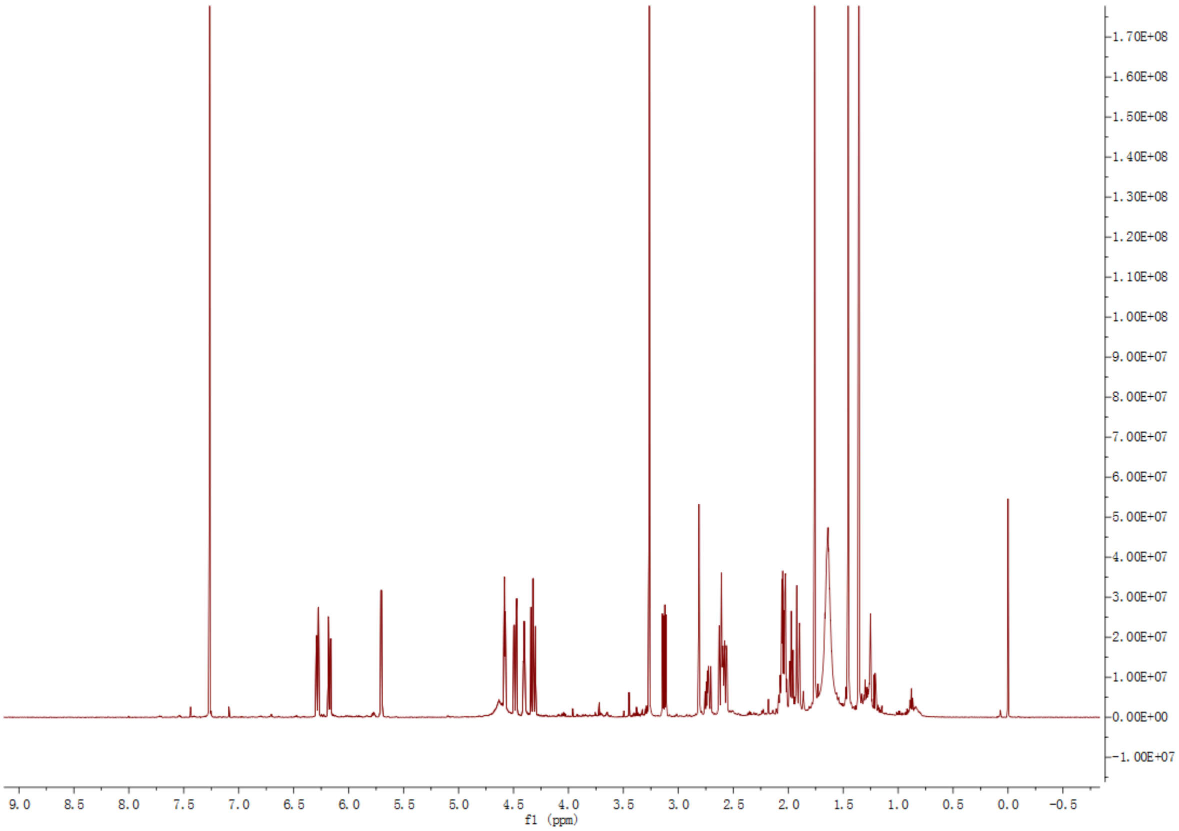

Fig. 13 ^{1}H NMR spectrum of compound **3** ($CDCl_3$, 600 MHz).

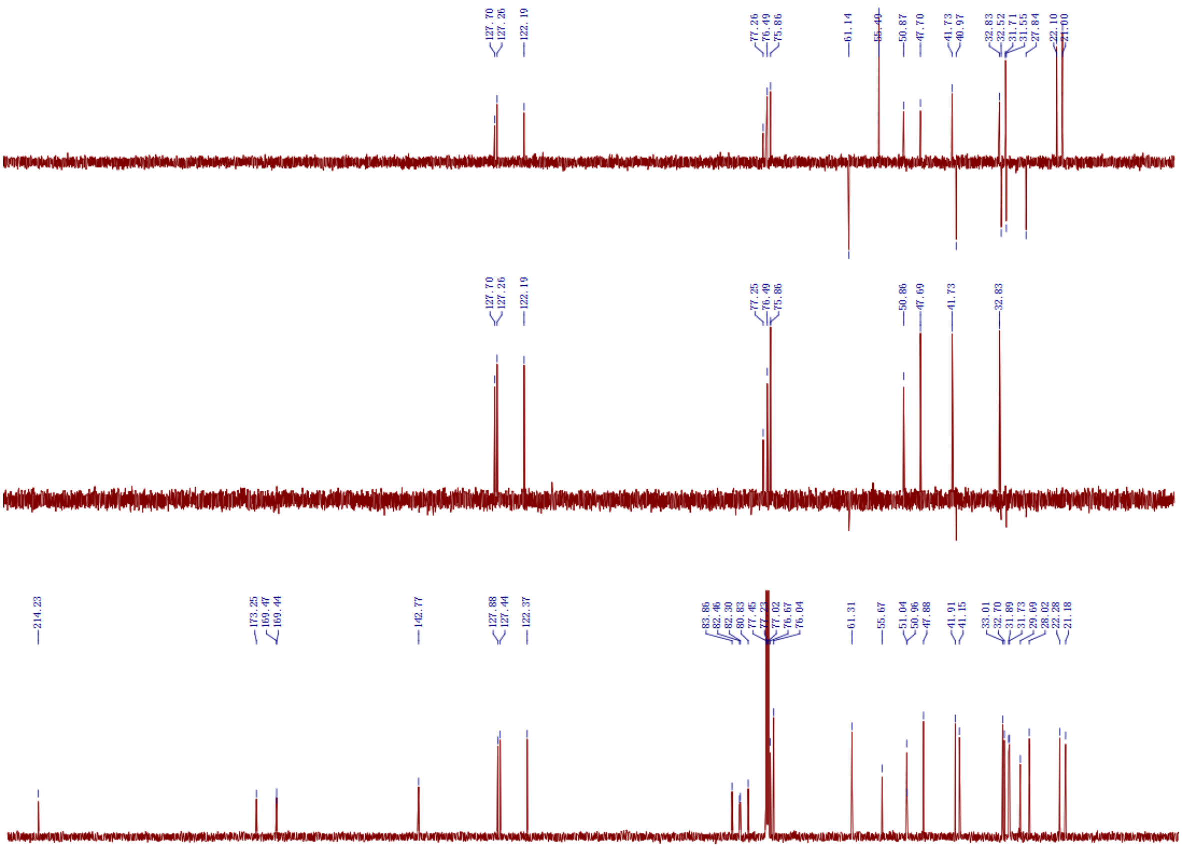

Fig. 14 ^{13}C NMR spectrum of compound **3** ($CDCl_3$, 150 MHz).

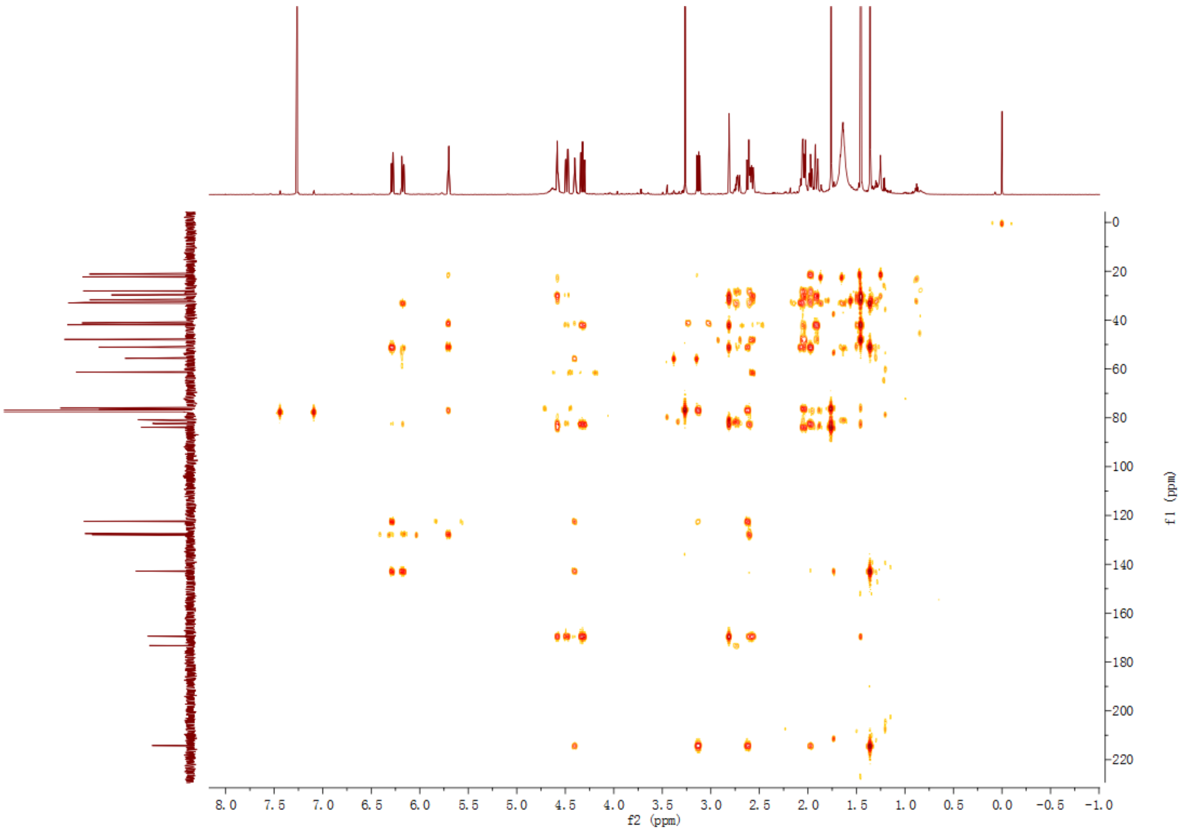

Fig. 15 HMBC spectrum of compound **3**.

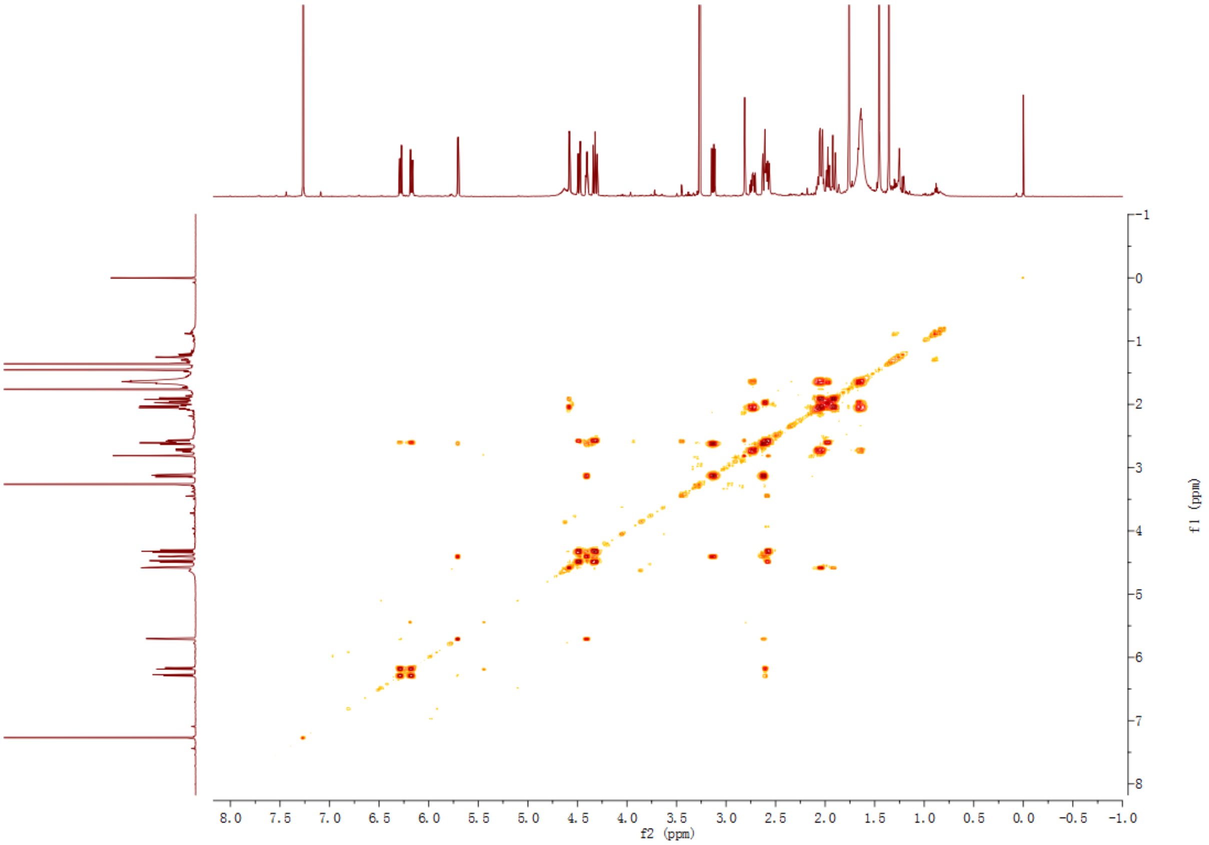

Fig. 16 COSY spectrum of compound **3**.

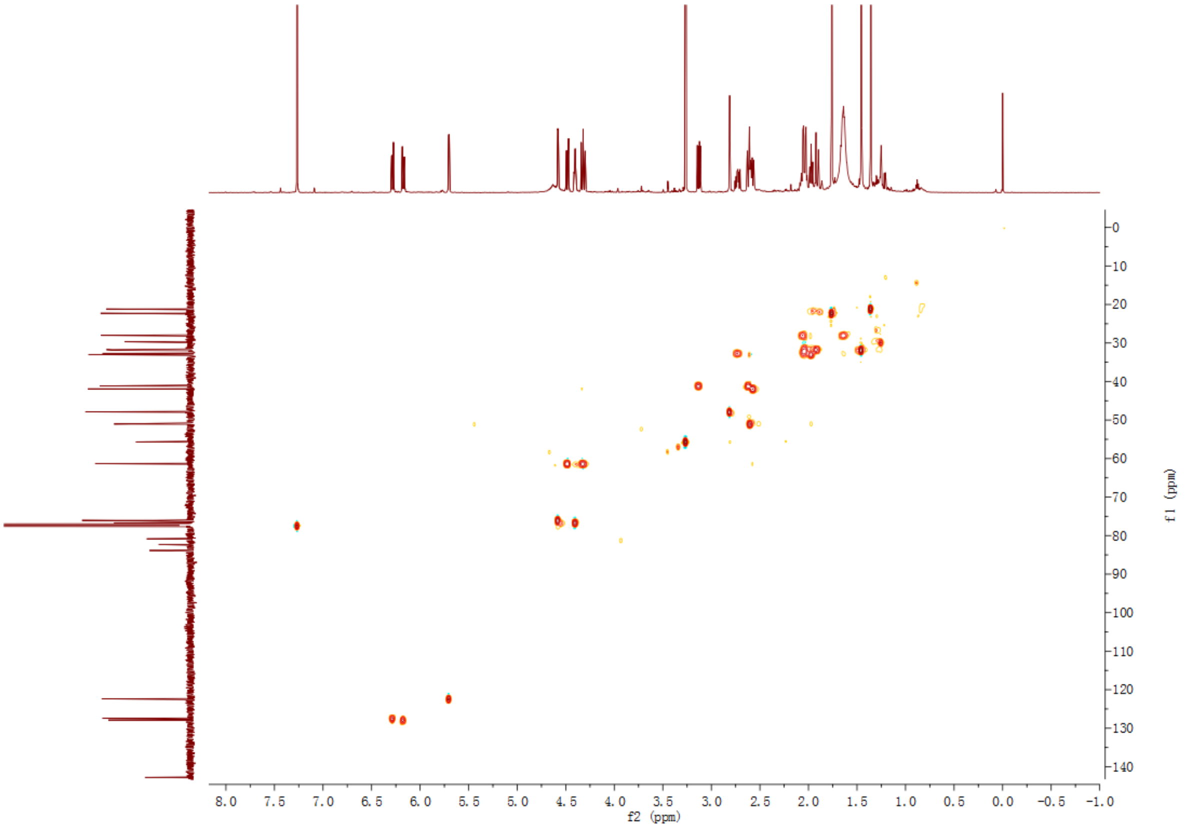

Fig. 17 HSQC spectrum of compound **3**.

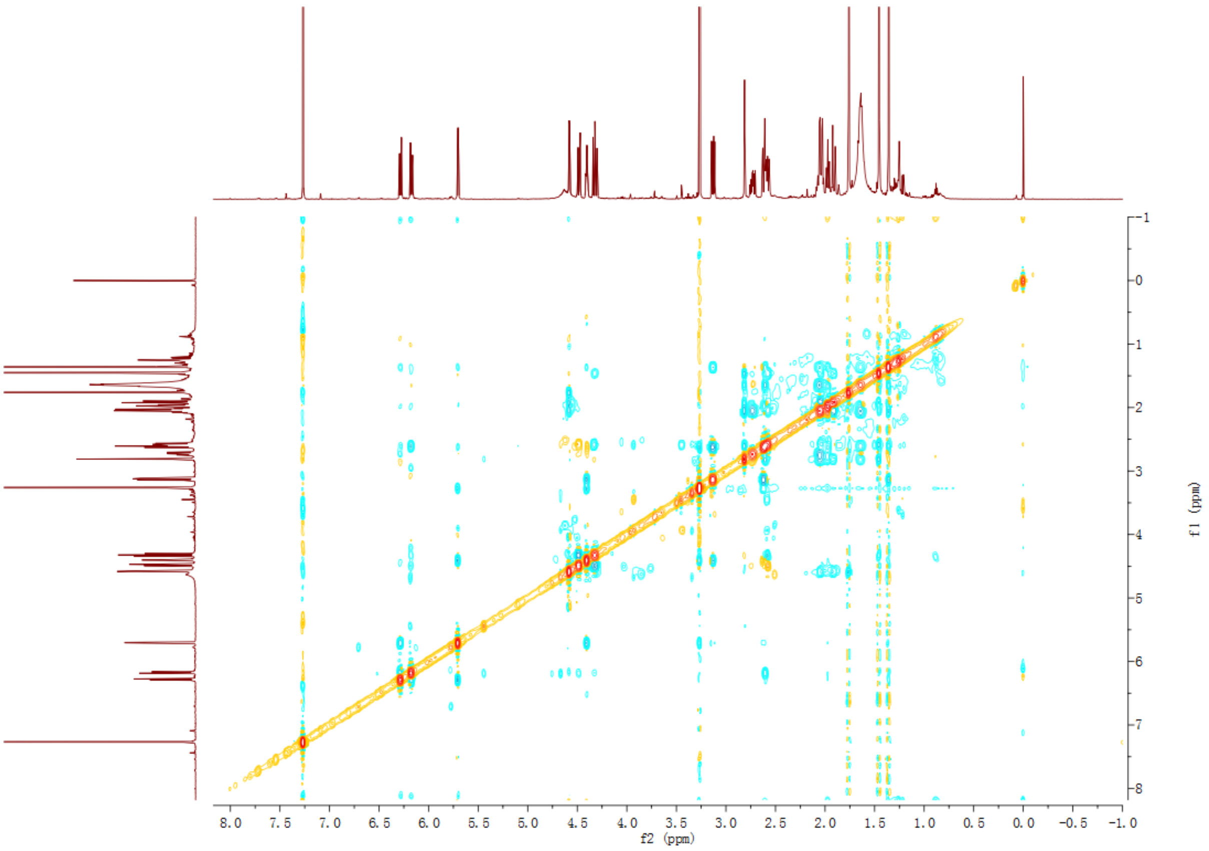

Fig. 18 ROESY spectrum of compound **3**.
